# Supplementary material for: COG6 is an essential host factor for influenza A virus infection
Source: Microbiol Spectr. 2025 Sep 5;13(10):e01362-25. doi: 10.1128/spectrum.01362-25 (PMC12502754; doi:10.1128/spectrum.01362-25)
Supplement: Supplemental Material — Figures S1 to S3 and Table S1. [file spectrum.01362-25-s0002.docx]

**Supplemental Materials**

**
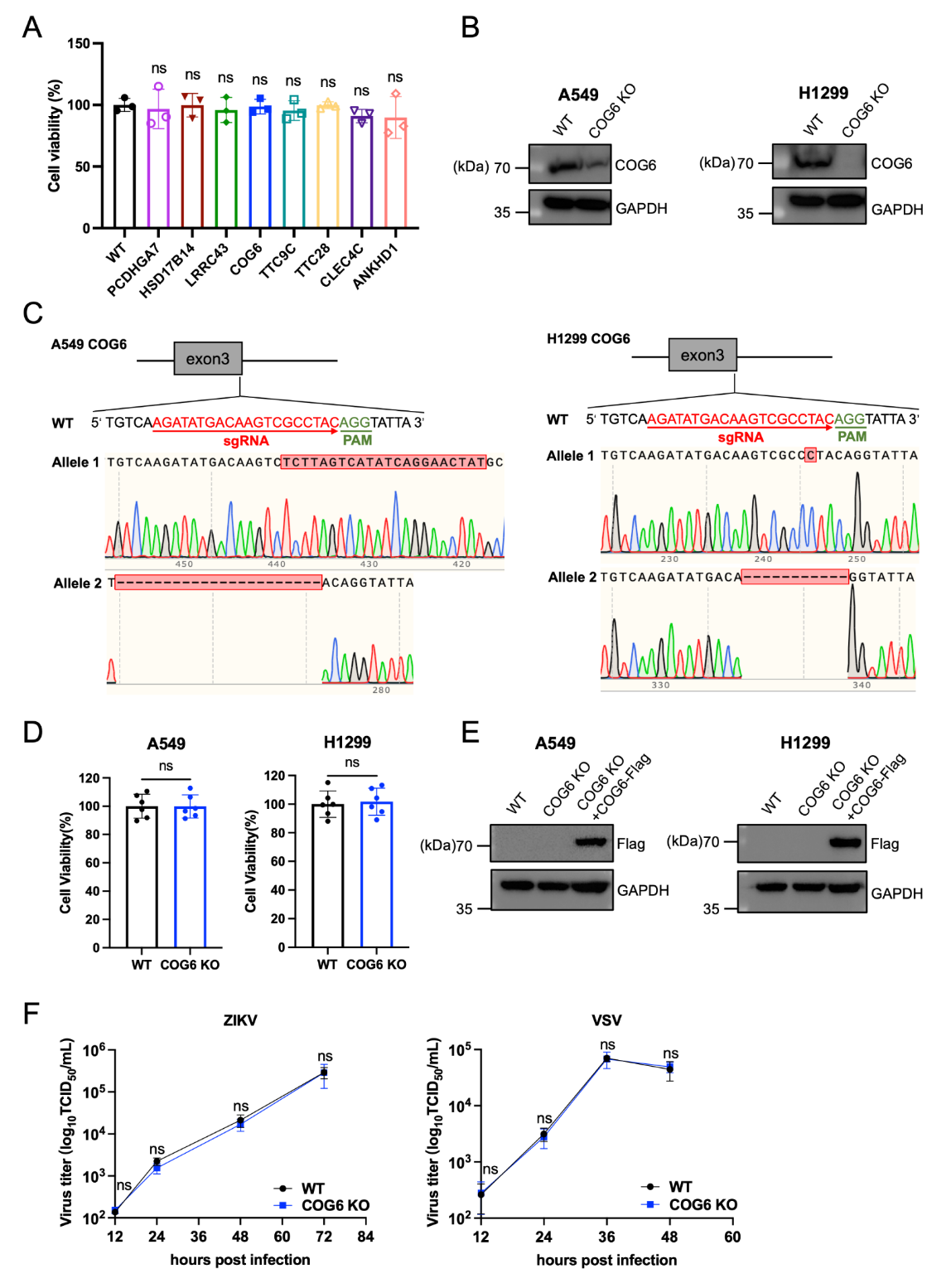
**

**FIG S1** Cell viability and validation of editing efficiency in A549 and H1299 cells by CRISPR sgRNA of genes selected. (**A**) Viability of A549 cells edited with individual CRISPR sgRNA of genes selected. An equal number of knockout polyclonal cells were plated and viability was assessed by CCK-8 assays. (**B** and **C**) Editing efficiency of COG6 knockout in A549 and H1299 cells. COG6 in the cells was edited by the indicated sgRNA and the monoclonal population of cells was subjected to western blotting (**B**) and sanger sequencing (**C**). (**D**) Viability of WT and COG6-KO A549 and H1299 cells was assessed by CCK-8 assays. (**E**) Western blot analysis showing lentiviral-mediated stable restoration of COG6 expression in COG6-KO cells. WT and COG6-KO cells were included as controls. GAPDH was used as a loading control. (**F**) WT and COG6-KO A549 cells were infected with ZIKV (MOI=0.5) or VSV-GFP (MOI=0.01). The supernatants were collected at indicated time points and virus titers were determined by TCID_50_ assay in A549 cells. The values displayed are the log_10_ mean ± SD from three biological replicates. Some error bars are too small to be clearly visible. (**A** and **D**) Data, presented as mean ± SD, are normalized to WT cells from three or six biological replicates. Statistical analyses were determined using one-way ANOVA with Dunnett’s multiple comparisons test in (**A**), or unpaired, two-tailed Student’s *t*-test in (**D** and **F**). *ns*, no significance.

**
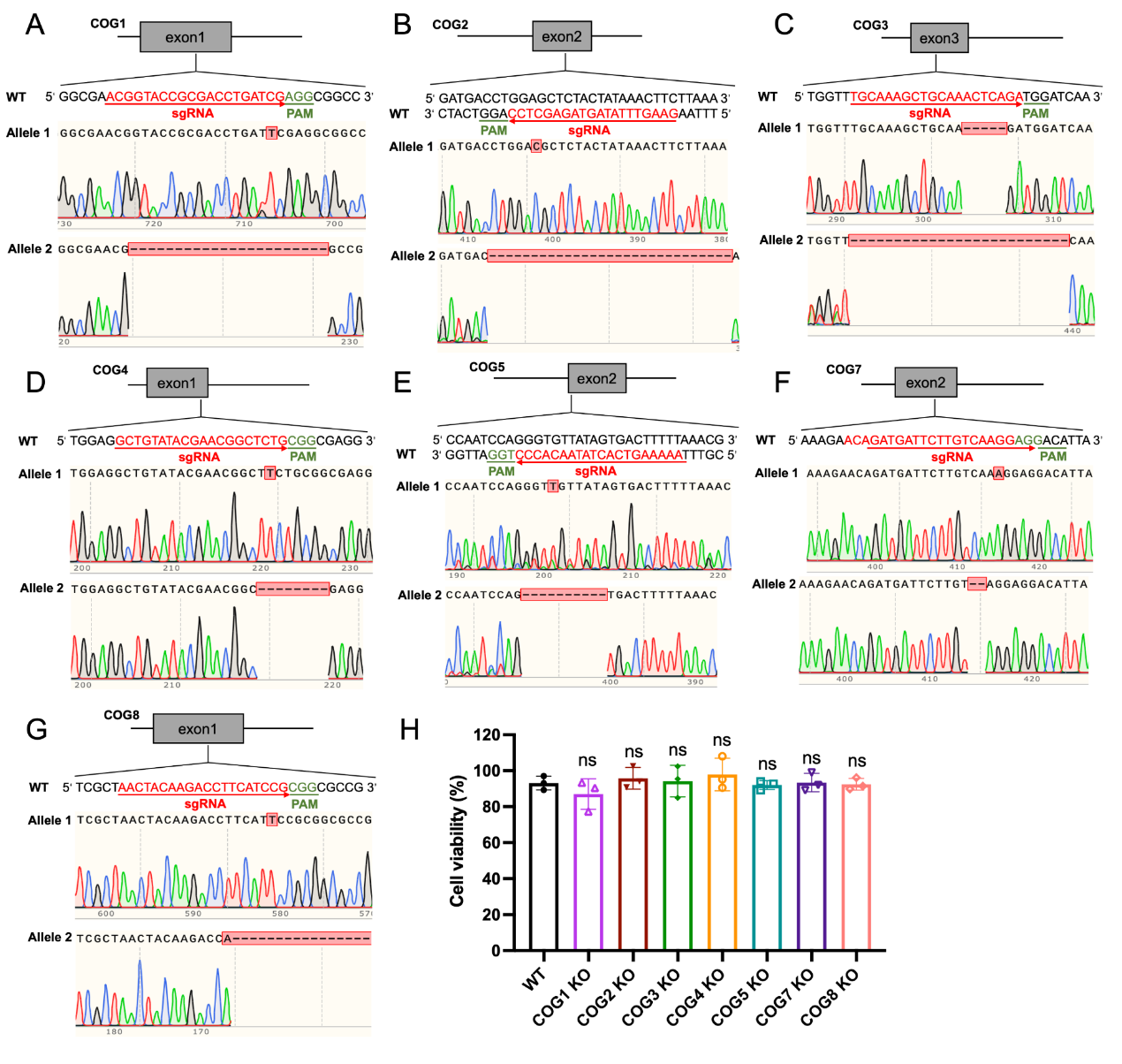
**

**FIG S2** Editing efficiency of H1299 cells by CRISPR sgRNA of genes selected, including COG1, COG2, COG3, COG4, COG5, COG7 or COG8. (**A-G**) COG1 (**A**), COG2 (**B**), COG3 (**C**), COG4 (**D**), COG5 (**E**), COG7 (**F**) or COG8 (**G**) in H1299 cells was edited by the indicated sgRNA and the monoclonal population of cells was subjected to sanger sequencing. (**H**) Viability of WT and COG1-KO, COG2-KO, COG3-KO, COG4-KO, COG5-KO, COG7-KO and COG8-KO H1299 cells was assessed by CCK-8 assays. Statistical analyses were from three biological replicates and determined using unpaired, two-tailed Student’s *t*-test in (**H**). *ns*, no significance.

**
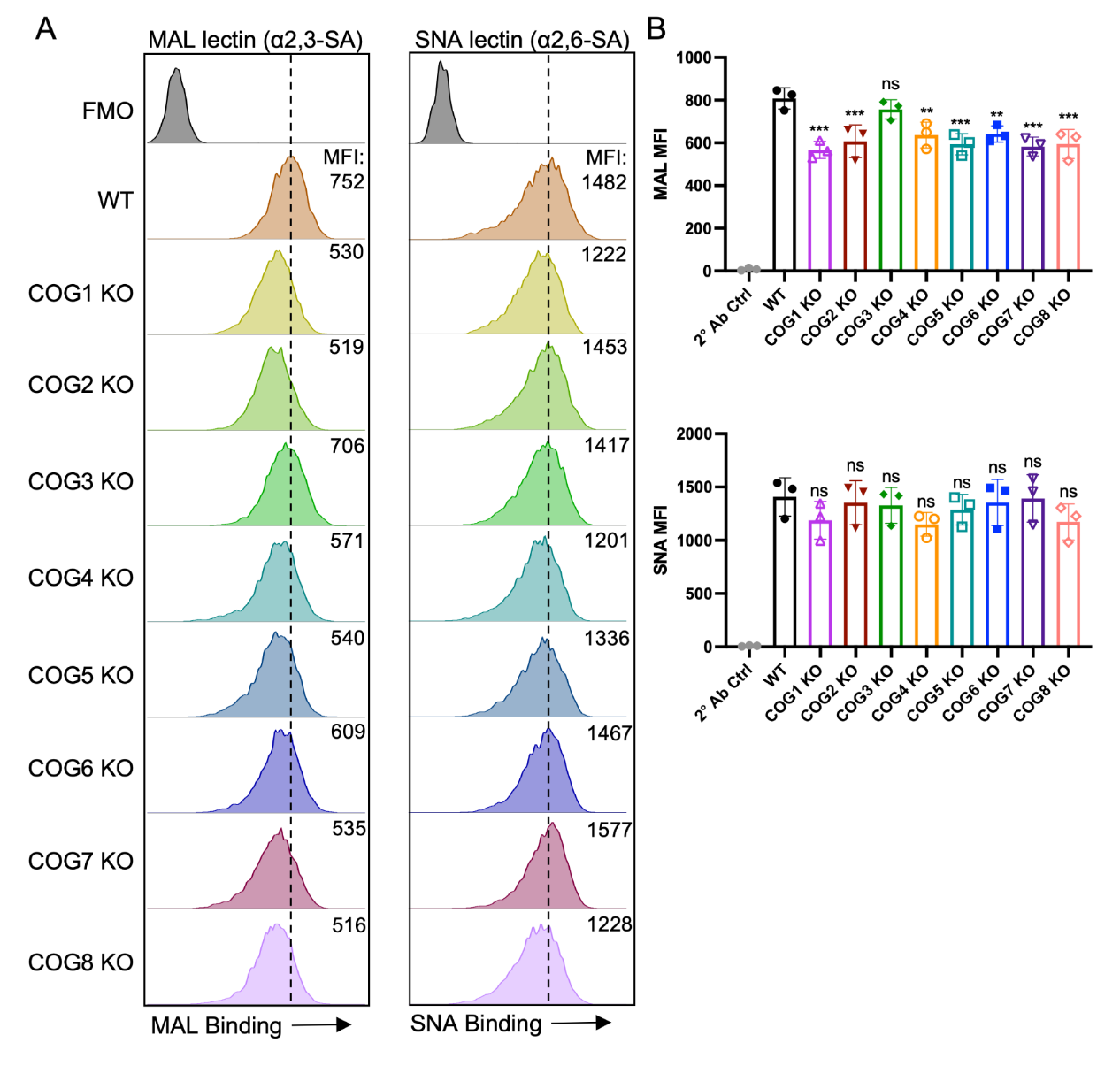
**

**FIG S3** Analyses of sialic acid expression by lectin staining. (**A** and **B**) Levels of α2,3- and α2,6-linked sialic acids on WT, COG1-KO, COG2-KO, COG3-KO, COG4-KO, COG5-KO, COG6-KO, COG7-KO or COG8-KO H1299 cells were stained with biotinylated MAL or SNA lectin. Lectins was detected using PE-labelled streptavidin. Representative histograms from three independent experiments are shown. Statistical analyses were determined using one-way ANOVA with Dunnett’s multiple comparisons test in (**B**). **, *p*<0.01; ***, *p*<0.001; *ns*, no significance.

**Table S1** List of primers.

| Experiments | Primer name | Primer sequence |
| --- | --- | --- |
| sgRNA synthesis | PCDHGA7-F | CACCGCACGGTACATGCTATCGACC |
| sgRNA synthesis | PCDHGA7-R | AAACGGTCGATAGCATGTACCGTGC |
| sgRNA synthesis | HSD17B14-F | CACCGCATATGGTGTCCGAGTCAAC |
| sgRNA synthesis | HSD17B14-R | AAACGTTGACTCGGACACCATATGC |
| sgRNA synthesis | LRRC43-F | CACCGTCTACGTCACCGCTAATCAC |
| sgRNA synthesis | LRRC43-R | AAACGTGATTAGCGGTGACGTAGAC |
| sgRNA synthesis | COG6-F | CACCGAGATATGACAAGTCGCCTAC |
| sgRNA synthesis | COG6-R | AAACGTAGGCGACTTGTCATATCTC |
| sgRNA synthesis | TTC9C-F | CACCGTCCGGCCCGATACAAGGCCT |
| sgRNA synthesis | TTC9C-R | AAACAGGCCTTGTATCGGGCCGGAC |
| sgRNA synthesis | TTC28-F | CACCGCCAGTGCCTTGTCATACTGC |
| sgRNA synthesis | TTC28-R | AAACGCAGTATGACAAGGCACTGGC |
| sgRNA synthesis | CLEC4C-F | CACCGTGCCAATGTCGCCGACCCCC |
| sgRNA synthesis | CLEC4C-R | AAACGGGGGTCGGCGACATTGGCAC |
| sgRNA synthesis | ANKHD1-F | CACCGGACGGGTCTTACCCCCTTGA |
| sgRNA synthesis | ANKHD1-R | AAACTCAAGGGGGTAAGACCCGTCC |
| sgRNA synthesis | COG1-F | CACCGACGGTACCGCGACCTGATCG |
| sgRNA synthesis | COG1-R | AAACCGATCAGGTCGCGGTACCGTC |
| sgRNA synthesis | COG2-F | CACCGGAAGTTTATAGTAGAGCTCC |
| sgRNA synthesis | COG2-R | AAACGGAGCTCTACTATAAACTTCC |
| sgRNA synthesis | COG3-F | CACCGTGCAAAGCTGCAAACTCAGA |
| sgRNA synthesis | COG3-R | AAACTCTGAGTTTGCAGCTTTGCAC |
| sgRNA synthesis | COG4-F | CACCGGCTGTATACGAACGGCTCTG |
| sgRNA synthesis | COG4-R | AAACCAGAGCCGTTCGTATACAGCC |
| sgRNA synthesis | COG5-F | CACCGAAAAAGTCACTATAACACCC |
| sgRNA synthesis | COG5-R | AAACGGGTGTTATAGTGACTTTTTC |
| sgRNA synthesis | COG7-F | CACCGACAGATGATTCTTGTCAAGG |
| sgRNA synthesis | COG7-R | AAACCCTTGACAAGAATCATCTGTC |
| sgRNA synthesis | COG8-F | CACCGAACTACAAGACCTTCATCCG |
| sgRNA synthesis | COG8-R | AAACCGGATGAAGGTCTTGTAGTTC |
| RT-qPCR | NP-F | ACGGCTGGTCTGACTCACAT |
| RT-qPCR | NP-R | TCCATTCCGGTGCGAACAAG |
| RT-qPCR | GAPDH-F | CTGGGCTACACTGAGCACC |
| RT-qPCR | GAPDH-R | AAGTGGTCGTTGAGGGCAATG |

**Supplementary Data 1** CRISPR screening. This Excel file contains the sgRNA-level analysis results from a genome-wide CRISPR/Cas9 knockout screen. It includes normalized read counts for each sgRNA in mock-treated (control) and virus-infected (treatment) cell populations. For each gene, log_2_ fold changes (LFC), *p*-values, and false discovery rates (FDR) were calculated based on sgRNA abundance. In addition, each gene is assigned an overall screening score.
